# Mechanism of Wnt signaling induced down regulation of *mrhl* long non-coding RNA in mouse spermatogonial cells

**DOI:** 10.1093/nar/gkv1023

**Published:** 2015-10-07

**Authors:** Vijay Suresh Akhade, Shrinivas Nivrutti Dighe, Shubhangini Kataruka, Manchanahalli R. Satyanarayana Rao

**Affiliations:** From the Molecular Biology and Genetics Unit, Jawaharlal Nehru Centre for Advanced Scientific Research, Jakkur P.O., Bangalore 560064, India

## Abstract

Long non coding RNAs (lncRNAs) have emerged as important regulators of various biological processes. LncRNAs also behave as response elements or targets of signaling pathway(s) mediating cellular function. Wnt signaling is important in regulating mammalian spermatogenesis. *Mrhl* RNA negatively regulates canonical Wnt pathway and gets down regulated upon Wnt signaling activation in mouse spermatogonial cells. Also, *mrhl* RNA regulates expression of genes pertaining to Wnt pathway and spermatogenesis by binding to chromatin. In the present study, we delineate the detailed molecular mechanism of Wnt signaling induced *mrhl* RNA down regulation in mouse spermatogonial cells. *Mrhl* RNA has an independent transcription unit and our various experiments like Chromatin Immunoprecipitation (in cell line as well as mouse testis) and shRNA mediated down regulation convincingly show that β-catenin and TCF4, which are the key effector proteins of the Wnt signaling pathway are required for down regulation of *mrhl* RNA. We have identified Ctbp1 as the co-repressor and its occupancy on *mrhl* RNA promoter depends on both β-catenin and TCF4. Upon Wnt signaling activation, Ctbp1 mediated histone repression marks increase at the *mrhl* RNA promoter. We also demonstrate that Wnt signaling induced *mrhl* RNA down regulation results in an up regulation of various meiotic differentiation marker genes.

## INTRODUCTION

Recent advancements in high throughput transcriptome analyses have revealed that most of the mammalian genome is transcribed to generate a huge repertoire of RNA molecules, among which long non coding RNAs (lncRNAs) are becoming increasingly important in cellular regulation. Long non-coding RNAs (lncRNAs) are a subclass of non-coding RNAs, generally greater than 200bp in length and could be sense, antisense, intronic or intergenic (1). LncRNAs are also known to regulate various important biological processes like genomic imprinting (*Air* and *Kcnq1ot1*) (2,3), dosage compensation (*Xist* and *roX*) (4,5), cell differentiation, development (*Fendrr, Bvht, Miat, Hotair*, etc.) (6), pluripotency (*Evx1as* and *Hoxb5/6as*) (7), nuclear architecture (*Neat1*) (8), etc. Since lncRNAs play such a diverse role in development and cellular differentiation, it is not surprising that they have employed multiple molecular mechanisms to exert their biological functions. These mechanisms include translational inhibition (lincRNA-p21) (9), mRNA degradation (*1/2-sbs* RNAs) (10), RNA decoys (*Gas5*) (11), facilitating recruitment of chromatin modifiers (*Mistral*, *HOTTIP*) (12,13), regulation of protein activity (*Evi2* and *Lethe*) (14,15), regulating the availability of miRNAs by sponging mechanism (linc-MD1) (16) etc. In addition to their role in cellular differentiation and development, expression of lncRNAs are also perturbed in several diseases and more particularly in cancer (17) suggesting that regulation of their expression is an important aspect of cellular homeostasis. Interestingly, most of the lncRNAs are transcribed by RNA Polymerase II and the chromatin architecture of lncRNA genes has revealed chromatin signatures characteristic of RNA polymerase II transcribed protein coding genes (18).

*Mrhl* lncRNA, discovered in our laboratory, is encoded in a meiotic recombination hot spot locus of the mouse chromosome 8, in the 15th intron of the *phkb* gene and transcribed by RNA Polymerase II (19). The primary transcript is 2.4 kb in length and is a nuclear restricted, polyadenylated and unspliced RNA (20). We had shown earlier that siRNA mediated down regulation of *mrhl* RNA in Gc1-Spg cells (spermatogonial cell line) activated Wnt signaling suggesting that *mrhl* RNA acts as a negative regulator of Wnt signaling in spermatogonial cells (21). Ddx5/p68 RNA helicase is an interacting partner of *mrhl* RNA in the nucleus and is required for the regulatory function of *mrhl* RNA. In a more recent study, we have mapped the chromatin occupancy of *mrhl* RNA and shown that *mrhl* RNA regulates the expression of several genes in a Ddx5/p68 dependent manner (22). Among these genes, many are known to play key roles in spermatogenesis as well as Wnt signaling. These experiments carried out in Gc1-Spg cells were also validated in the mouse testicular tissue. One of the key observations from these studies was that Wnt3a ligand triggers Wnt signaling in Gc1-Spg cells and at the same time down regulates *mrhl* RNA gene expression (21). It is now well documented that, Wnt signaling is repressed in spermatogonial cells and is activated in meiotic spermatocytes (23,24). Interestingly, expression of *mrhl* RNA is highly down regulated in spermatocytes in comparison to spermatogonial cells (22). This raises the possibility that *mrhl* RNA may play a significant role in meiotic commitment of spermatogonial cells. In this context, we have now carried out a detailed molecular analysis of the mechanism underlying the Wnt3a ligand mediated down regulation of *mrhl* RNA gene in spermatogonial cells.

In a series of experiments, we show that *mrhl* RNA gene is a target of Wnt signaling and its Wnt3a triggered down regulation is mediated by the occupancy of β-catenin at the TCF4 binding site present in the upstream promoter region of the gene and dependent upon the recruitment of the co-repressor Ctbp1 at this promoter region. The down regulation of *mrhl* RNA gene expression is associated with the establishment of repressive chromatin signature at the proximal promoter region. Interestingly, we also find that the markers of meiotic commitment or meiotic progression are up regulated following Wnt3a ligand treatment of Gc1-Spg cells and the down regulation of *mrhl* RNA upon Wnt3a ligand treatment is essential for the up regulation of these marker gene expression.

## MATERIALS AND METHODS

### Cell line, antibodies, plasmids, other chemicals

Gc1-Spg cell line (ATCC, CRL-2053) and HEK293 (ATCC, CRL-1573) were maintained as described previously (22). Mouse L-control cell line (ATCC, CRL-2648) and L-Wnt3A cell line (ATCC, CRL-2647) were kind gifts from Dr Jomon Joseph (NCCS, India).

The list of antibodies used in the present study is given below with their manufacturer and catalogue number in brackets: β-catenin (BD Transduction, 610154), TCF4 (Millipore, 05-511), Phospho S9 Gsk3β (Cell Signaling, 9336), Phospho S33/S37/T41 (Cell Signaling, 9561), Histone H3 (Abcam, ab6002), Alpha tubulin (Sigma–Aldrich, T8203), GAPDH (Abcam, ab8245), Ctbp1 (Abcam, ab129181), Chd8 (Abcam, ab84527), G9a (Cell Signaling, 3306), p300 (Sigma–Aldrich, P2859), Hdac1 (Cell Signaling, 5356), Hdac2 (Cell Signaling 5113), H3K9ac (Abcam, ab4441), H3K9me3 (Abcam, ab8893), H3K14ac (Abcam, ab52946), RFP (Abcam, ab62341), Lamin B (Santa Cruz Biotechnology, sc-6217), Scp3 (Abcam, ab97672).

The list of plasmids from Addgene with their plasmid ID and name of the depositor is given in brackets: dnTCF4 (16513, Bert Vogelstein), pcDNA3-β-catenin (16828, Eric Fearon), β-catenin shRNA (19761, William Hahn), scrambled shRNA (1864, David Sabatini). Following plasmids were obtained from Sigma–Aldrich and their catalogue number is given in brackets: TCF4 shRNA (TRCN0000012094), Ctbp1 shRNA (TRCN0000085774), TCF4 shRNA targeting 3′ UTR (TRCN0000012093), β-catenin shRNA targeting 3′ UTR (TRCN0000012688), Ctbp1 shRNA targeting 3′ UTR (TRCN0000085773). Following plasmids were generous gifts from Prof. Sanjeev Galande (IISER Pune, India): RFP tagged constructs of full length β-catenin (β-cat FL-RFP), N terminal β-catenin (β-cat N-RFP), C- terminal β-catenin (β-cat C-RFP), GST tagged full-length β-catenin (β-cat FL-GST), Flag tagged TCF4 construct (Flag-TCF4) and Flag tagged Ctbp1 plasmid construct (Flag-Ctbp1) were kind gifts from Prof. G. Chinnadurai (Saint Louis University, School of Medicine). Plasmids were used for transient transfection in Gc1-Spg cells at a final concentration of 1.5 μg/ml using Lipofectamine 2000 as per manufacturer's instructions.

All chemical reagents were of AR grade and were purchased from Sigma. Protein A agarose beads (15918-014) and Lipofectamine 2000 (11668027) were purchased from Invitrogen. DNAseI (MO303), NheI (R0131S), BamHI (R0136S) and XhoI (R0146S) were purchased from New England Biolabs. Luciferase assay Kit was supplied by Promega, Streptavidin HRP (ab 7403) obtained from Abcam and Recombinant TCF4 protein from Origene (TP760702)

#### Preparation of control and Wnt3a conditioned medium

L-control or L-Wnt3a cells (1 × 10^6^ cells) were seeded in 90mm culture dishes containing DMEM with 10% FBS. After 48 h, the cells were cultured with fresh medium and incubated for 1 more day. The medium was then collected, centrifuged at 500 × g for 5min, filtered through a 0.2-μm syringe filter and subsequently stored at −20°C till further use. For treatment of Gc1-Spg cells, the control as well as Wnt3a conditioned medium were diluted 2:1 with DMEM containing 10%FBS.

#### Cloning of 2.4kb mrhl RNA gene, mrhl RNA promoter and Luciferase assay

The 2.4 kb *mrhl* RNA gene was cloned in pcDNA 3.1 mammalian expression vector in between BamHI and XhoI sites. Clones were confirmed by sequencing. The 1 kb sequence upstream to *mrhl* RNA gene was cloned in pGL3-Basic vector in between NheI and XhoI restriction enzyme sites. Clones were confirmed by sequencing. The TCF4 binding site on *mrhl* RNA promoter (−175 bp) was mutated from ATCAAAG to ATCAGCG using Stratagene QuikChange Site-Directed Mutagenesis Kit (catalog #200518). For Luciferase assay, Gc1-Spg cells were transfected with 1 μg of the pGL3-Basic vector or 1 μg of *mrhl* RNA promoter clone in a six-well plate using Lipofectamine 2000. Transfection with CMV-βGal (0.4 μg) plasmid was done as an internal control for transfection efficiency. After 24 h, cells were harvested and processed for Luciferase assay as per the manufacturer's protocol (Promega). Luciferase readings for the pGL3- Basic as well as *mrhl* RNA promoter plasmid were normalized with the Luminometer readings obtained for CMV-βGal.

#### Electrophoretic mobility shift assay (EMSA)

Equimolar amounts of biotinylated complementary oligos were annealed at 95°C for 5 min. Binding was set for both wild type and mutant oligonucleotides with 2 μg and 4 μg of TCF4 recombinant protein in buffer containing 10% glycerol, 5 mM MgCl_2_, 2% NP40, salmon sperm DNA and 1× binding buffer (10× binding buffer—250 mM Tris, 800 mM NaCl, 350 mM KCl, 10 mM DTT). The reaction mixture was incubated at room temperature for 1 h. Samples were resolved in 5% native polyacrylamide gel with 0.5× TBE as running buffer. Transfer was done in 0.5× TBE at 380 mA for 45 min followed by which the Nylon membrane was exposed to UV light for 10 min. Then the blot was subjected to 40 min of blocking (3% BSA in 1× TBS) followed by 40 min in streptavidin conjugated HRP (1:1000 dilution in 1% BSA in 1× TBS). After two washes of 10 min each in 1× TBS the blot was analyzed.

### Preparation of meiotic spreads and RNA FISH

In order to obtain the pool of different stages of spermatogenic cells, we selected 7, 14, 16, 18 and 20 days old mice, testes were dissected out, chopped in DMEM medium and filtered through cheese cloth. Cells were fixed in 4% PFA for 15 min at room temperature with gentle agitation. Later, cells were washed once with PBS, permeabilized with 0.5% Triton X-100 in PBS for 10 min with gentle agitation and again washed with PBS at room temperature. Cells were suspended in appropriate amount of PBS, spread over the cover slip and were allowed to dry in humidified chamber for 2–3 h.

For RNA-FISH experiment, we used Cy5 labeled probe (Custom LNA oligonucleotide, 5′-end labeled, Probe Sequence—cagctaggccaagacaacaaaatg procured form Exiqon) against *mrhl* RNA (20) and hybridization was carried out as described by de Planell-Saguer *et al*. (25) with minor modifications. Cells were blocked by incubating in prehybridization buffer (3% bovine serum albumin and 4× saline–sodium citrate buffer [SSC]) for 1 h at room temperature. Meanwhile, hybridization mix (25nM LNA-Cy5 probe in 10× dextran sulfate and 4× SSC) was prewarmed and hybridization was carried out at 50°C (20–25°C below the predicted probe *T*_m_) for 1 h with gentle agitation. To remove nonspecific binding of the probe, cells were washed with washing buffer-I (4× SSC, 0.1% Tween-20) three times for 5 min each at room temperature. Subsequently, cells were washed once for 5 min each with washing buffer-II (2× SSC), washing buffer-III (1× SSC), PBS and processed for immunofluorescence staining. Cells were blocked in 1% BSA for 45 min at 4°C and subsequently incubated at room temperature with primary antibody (anti-β-catenin 1:150 & anti-SCP3 1:200, diluted with 0.1% BSA) for 30 min. Cells were washed three times with PBS for 5 min each, followed by incubation with appropriate Alexa Fluor conjugated secondary antibody (1:400 diluted with 0.1% BSA) for 30 min at room temperature. The cells were washed thrice for 5 min each with PBS and nuclear stained with 1 μg/ml 4′,6′-diamidino-2-phenylindole (DAPI) and mounted in 60% glycerol. The images were acquired in an LSM 10 Meta Confocal microscope (Carl Zeiss) and analyzed by the software provided by Carl Zeiss and intensity quantification was done using ImageJ software.

#### Chromatin immunoprecipitation (ChIP) and sequential ChIP

ChIP was carried out as described previously (21), with a minor modification, wherein the genomic DNA was sonicated to obtain enrichment in a range from 100 to 300 bp. For sequential ChIP or Re-ChIP the procedure was followed as described by Gannon *et al*. (26) with few modifications. For Re-ChIP, the elution of complexes during the first ChIP was carried using 50 μl of 10 mM DTT at 37°C for 40 min. After elution the beads were separated and the elute was diluted 20 times with Re-ChIP buffer [1% Triton X-100, 2 mM EDTA, 150 mM NaCl, 20 mM Tris–HCl, (pH 8.1)] and subjected again to the ChIP procedure. For ChIP and Re-ChIP with P7 and P21 testes, whole mice testes were used. In ChIP experiments, the fold enrichment over input was calculated considering the percentage of input chromatin used for ChIP and the Ct values obtained for the target from Input DNA and ChIP DNA. The calculations were done as follows:

\begin{equation*} \begin{array}{*{20}l} {{\rm Fold}\;{\rm enrichment}\;{\rm over}\;{\rm input} = } \\ {\% \;{\rm of}\;{\rm input} \times {2^ {\wedge}} [{\rm Ct}({\rm input}) - {\rm Ct}({\rm ChIP})]} \\ \end{array} \end{equation*}

## RESULTS

### Down regulation of *mrhl* RNA expression upon Wnt3a conditioned medium treatment in Gc1-Spg cells

**Figure 1.**
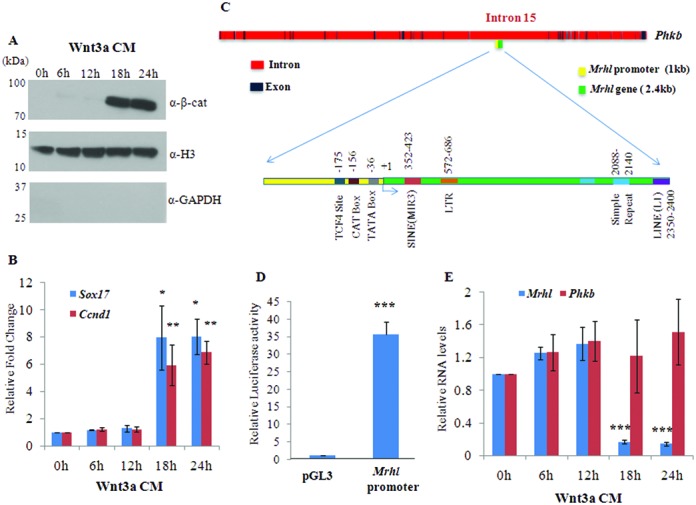
Down regulation of *mrhl* RNA upon Wnt3a CM treatment in Gc1-Spg cells. Mouse spermatogonial Gc1-Spg cells were treated with Wnt3a CM for different time durations (0, 6, 12, 18 and 24 h). (**A**) Western blot analysis (using nuclear lysate) for β-catenin. Presence of histone H3 and absence of GAPDH shows purity of nuclear fractions. (**B**) Expression analysis of *Sox17* and *Ccnd1* which are known targets of Wnt signaling during spermatogenesis. (**C**) Genomic organization of *mrhl* RNA gene embedded within the 15th intron of *phkb* gene. The locations of TATA box, CAT box and TCF4 binding site are depicted in the 1 kb upstream promoter region of *mrhl* RNA gene while different repeat elements are shown in the body of *mrhl* RNA gene. (**D**) Luciferase activity of the 1kb upstream promoter region of *mrhl* RNA gene. (**E**) Expression analysis of *mrhl* RNA and *phkb* RNA upon Wnt3a CM treatment. For (B), (D) and (E) the data are plotted as mean ± SD, *n* = 4. ***P ≤ 0.0005, **P ≤ 0.005, *P ≤ 0.05 (*t*-test).

In our earlier report, we demonstrated the down regulation of *mrhl* RNA after Wnt3a ligand treatment of Gc1-Spg cells (21). As an initial step towards delineating the molecular mechanism of this down regulation, we monitored different intracellular steps of the signaling process as a function of time of Wnt3a ligand exposure (Wnt3a conditioned medium prepared using mouse L cells) leading to the down regulation of *mrhl* RNA gene expression. Activation of Wnt signaling was assessed by various parameters which included an increase in the inhibitory phosphorylation (Ser9) of Gsk3β, decrease in Ser33/Ser37/Thr41 phosphorylated form of β-catenin, nuclear translocation of β-catenin and activation of *Sox17* and *CyclinD1* gene expression (target genes of Wnt signaling pathway during spermatogenesis). Activation of Wnt signaling was observed at 18 h of Wnt3a CM treatment as monitored by the nuclear translocation of β-catenin (Figure 1A). Presence of histone H3 and absence of GAPDH confirmed the purity of the nuclear lysate preparation. Immunofluorescence experiment also showed the nuclear translocation of β-catenin at 18 h and 24 h of Wnt3a CM treatment (Supplementary Figure S1A or S1A). Western blot analysis using total cell lysates of Gc1-Spg cells showed the increase of phospho Ser9 Gsk3β and a decrease of phospho Ser33/Ser37/T41 β-catenin at 18 h of Wnt3a CM treatment (Supplementary Figure S1B or S1B). Furthermore, the increased expression of *Sox17* and *CyclinD1* was observed at 18 h and 24 h of Wnt3a CM treatment (Figure 1B). For comparative analysis, we have shown similar immunofluorescence analysis in HEK 293 cell line where β-catenin starts appearing in the nucleus from 6 h post-treatment of Wnt3a CM which is much earlier compared to Gc1-Spg cells. Thus, the kinetics of Wnt signaling activation seems to be cell line dependent.

*Mrhl* RNA is transcribed from the 15th intron of *phkb* gene and the genomic organization of *mrhl* RNA gene is shown in Figure 1C. The 2.4 kb *mrhl* RNA gene contains various repeat elements like L1 LINE element, MIR3 SINE element, simple repeat and LTR element. The 1 kb upstream region of *mrhl* RNA gene contains eukaryotic RNA polymerase II promoter elements like the TATA box and the CAAT box. We also observe a consensus TCF4 binding site (A/T-A/T-C-A-A-A-G) at −175 bp position. Also, by bioinformatics analysis using TFM explorer we could find putative binding sites for many transcription factors like FoxA1, FoxA2, FoxQ1 and Nkx3-2. In order to validate whether the 1 kb upstream region can function as a promoter element, we cloned the 1kb genomic DNA sequence upstream of *mrhl* RNA gene in the pGL3 Basic vector and checked for the Luciferase activity of the DNA fragment in Gc1-Spg cells. Luciferase assay showed ∼35-fold increase in Luciferase activity compared to empty vector control, thus demonstrating the promoter activity of the 1 kb upstream DNA fragment (Figure 1D). We then checked the expression levels of *mrhl* RNA on Wnt3a CM treatment. Down regulation of *mrhl* RNA expression was observed at 18 h and 24 h of Wnt3a CM treatment (Figure 1E). Since *mrhl* RNA is transcribed from the intron of *phkb* gene, it was possible that the down regulation of *mrhl* RNA could be because of down regulation of *phkb* gene expression. For this, we also assessed the expression of *phkb* gene upon Wnt3a CM treatment. We did not observe any significant change in the *phkb* gene expression (Figure 1E); suggesting that indeed *mrhl* RNA was specifically down regulated upon Wnt3a CM treatment and hence behaves as an independent transcription unit. We have previously demonstrated that Wnt signaling mediated *mrhl* RNA down regulation is also relevant *in vivo* in mouse spermatogenic cells wherein *mrhl* RNA is significantly down regulated in 21-day-old mouse testis (P21 testis) which predominantly contain spermatocytes and represent Wnt activated state as opposed to 7-day-old mouse testis (P7 testis) which predominantly contain spermatogonia and represent Wnt repressed state (22). Therefore, in most of our subsequent experiments, we have used P7 and P21 mouse testis as an *in vivo* analogy to Gc1-Spg cells grown in control medium and Wnt3a CM respectively.

### β-Catenin and TCF4 occupy the proximal promoter of *mrhl* RNA gene upon Wnt signaling activation

**Figure 2.**
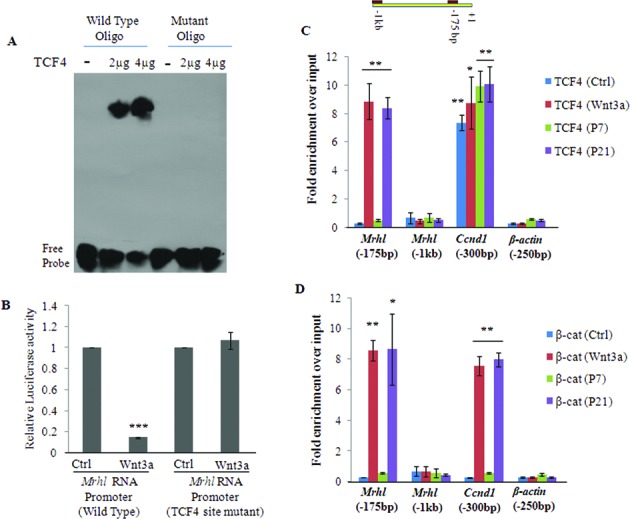
β-Catenin and TCF4 occupancy of the *mrhl* RNA promoter at the TCF4 binding site. (**A**) Electrophoretic mobility shift assay (EMSA) using TCF4 protein and DNA oligos with either a wild type or a mutant TCF4 binding site. Two different concentrations (2μg and 4μg) of TCF4 protein were used in the assay. (**B**) Luciferase assay in Gc1-Spg cells treated with control medium or Wnt3a CM using plasmid constructs containing 1 kb upstream promoter region of *mrhl* RNA gene with either wild type TCF4 binding site or a mutant TCF4 binding site. (**C** and **D**) TCF4 (C) and β-catenin (D) ChIP showing their occupancy on the *mrhl* RNA promoter region containing the TCF4 binding site [*Mrhl*(−175 bp)] selectively upon Wnt signaling activation. P7 testis represents Wnt repressed state while P21 testis represents Wnt activated state. Data in (B), (C) and (D) are plotted as mean ± SD, *n* = 4. ***P ≤ 0.0005, **P ≤ 0.005, *P ≤ 0.05 (*t*-test).

As mentioned above, the *mrhl* gene proximal promoter also contains a TCF4 binding site (A/T-A/T-C-A-A-A-G) at −175 position [further referred as *Mrhl*(−175 bp)]. TCF4 is a key transcription factor of Wnt signaling and its regulatory function is dependent on β-catenin. The β-catenin–TCF4 complex occupies the Wnt target gene promoters and brings about activation/repression based on the recruitment of either co-activators or co-repressors (27). Since the timing of down regulation of *mrhl* RNA and β-catenin nuclear translocation coincided (Figure 1A and Supplementary Figure S1A), we considered the possibility of β-catenin and TCF4 as the regulators of *mrhl* RNA gene expression upon activation of Wnt signaling by the Wnt3a ligand. Towards this objective, we initially carried out an *in vitro* EMSA using recombinant TCF4 protein and DNA oligonucleotides harboring either the wild type or mutant TCF4 binding site. We could detect a mobility shift only with the oligonucleotide containing a wild-type TCF4 binding site (Figure 2A). In order to determine if binding of TCF4 regulates the expression of *mrhl* RNA, we cloned the 1 kb upstream promoter sequence of *mrhl* RNA gene with site directed mutation in the TCF4 binding site and performed the Luciferase reporter assay in Gc1-Spg cells using control medium and Wnt3a CM. The reporter activity of the wild-type *mrhl* RNA promoter was substantially reduced upon treatment of Wnt3a CM while this reduction was not observed with the TCF4 site mutant *mrhl* RNA promoter construct (Figure 2B). This suggested that TCF4 binding regulates the expression of *mrhl* RNA only upon Wnt3a CM treatment. Subsequently, we carried out Chromatin Immuno-precipitation (ChIP) experiments using anti β-catenin and anti TCF4 antibodies in control medium treated and Wnt3a CM treated Gc1-Spg cells as well as in P7 and P21 mice testes. The data presented show the occupancy of TCF4 (Figure 2C) and β-catenin (Figure 2D) at *Mrhl* (−175bp) only upon Wnt signaling activation (Wnt3a-CM treated Gc1-Spg and P21 testis chromatin) but not in control medium treated Gc1-Spg or P7 testis chromatin. A 250 bp upstream region of *β-actin* promoter [*β-actin* (−250 bp)] and also the region from −800 bp to −1 kb within the *mrhl* RNA gene promoter [*Mrhl* (−1 kb)] which does not contain a TCF4 binding site were used as negative controls. As a positive control, we used the 300 bp upstream region of *CyclinD1* promoter [*Ccnd1*(−300 bp)] which contains a TCF4 binding site. The occupancy of TCF4 at *Ccnd1*(−300 bp) even in the absence of Wnt signaling activation (control medium treated Gc1-Spg and P7 testis) is consistent with the earlier reports demonstrating occupancy of TCF4 at subset of its target gene promoters even in the absence of Wnt signaling activation (28). These experiments collectively suggest that TCF4 and β-catenin occupy the proximal *mrhl* RNA promoter at −175 bp selectively upon activation of Wnt signaling.

### β-Catenin and TCF4 are required for down regulation of *mrhl* RNA upon Wnt signaling activation

**Figure 3.**
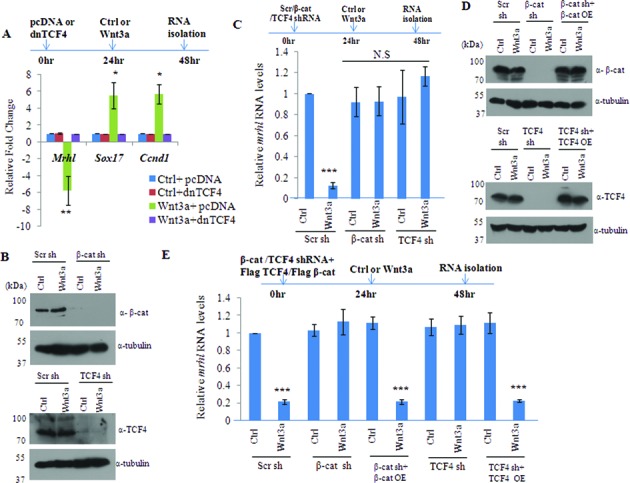
β-catenin and TCF4 are required for the down regulation of *mrhl* RNA upon Wnt signaling activation. (**A**) Over expression of dominant negative TCF4 (dnTCF4) with pcDNA as empty vector control and expression analysis of *mrhl* RNA. (**B**) Western blot showing drastic down regulation of β-catenin and TCF4 upon treatment of Gc1-Spg cells with β-catenin shRNA and TCF4 shRNA respectively. (**C**) Expression analysis of *mrhl* RNA upon treatment with β-catenin shRNA or TCF4 shRNA. (**D**) Expression analysis of β-catenin and TCF4 by western after treatment of Gc1-Spg cells with shRNAs targeting the UTR region of β-catenin or TCF4 mRNA as well as after over expression/rescue of β-catenin or TCF4 protein. (**E**) Expression analysis of *mrhl* RNA upon treatment with shRNAs targeting the 3′ UTR region of β-catenin or TCF4 mRNA as well as after over expression/rescue of β-catenin or TCF4 protein. Data in (A), (C) and (E) are plotted as mean ± SD, *n* = 4. ***P ≤ 0.0005, **P ≤ 0.005, *P ≤ 0.05 (*t*-test). N.S.: not significant.

Occupancy of β-catenin and TCF4 at the Wnt responsive elements (WRE) of *mrhl* RNA gene promoter suggested the possible role of β-catenin and TCF4 in down regulation of *mrhl* RNA upon Wnt signaling activation. In order to test this possibility, we first examined whether the interaction of β-catenin and TCF4 is essential for *mrhl* RNA down regulation by over-expression of the dominant negative form of TCF4 (dnTCF4) in Gc1-Spg cells. dnTCF4 is a truncated form of TCF4 with N-terminal deletion of the first 32 amino acids which renders TCF4 incapable of interacting with β-catenin (29). After 24 h of transfection with dnTCF4 containing plasmid, Gc1-Spg cells were treated with either control medium or Wnt3a CM for next 24 h and thereafter the expression of *mrhl* RNA, *Sox17* and *CyclinD1* were analyzed. As seen in Figure 3A, *mrhl* RNA was down regulated and *Sox17* and *CyclinD1* expression was up regulated after 24 h of Wnt3a CM treatment. However, upon dnTCF4 over expression and subsequent Wnt3a CM treatment, the expression levels of *mrhl* RNA, *Sox17* and *CyclinD1* were not perturbed (comparable to levels in control medium treated cells). This shows that physical interaction of β-catenin and TCF4 is required for the down regulation of *mrhl* RNA expression upon Wnt signaling activation.

**Figure 4.**
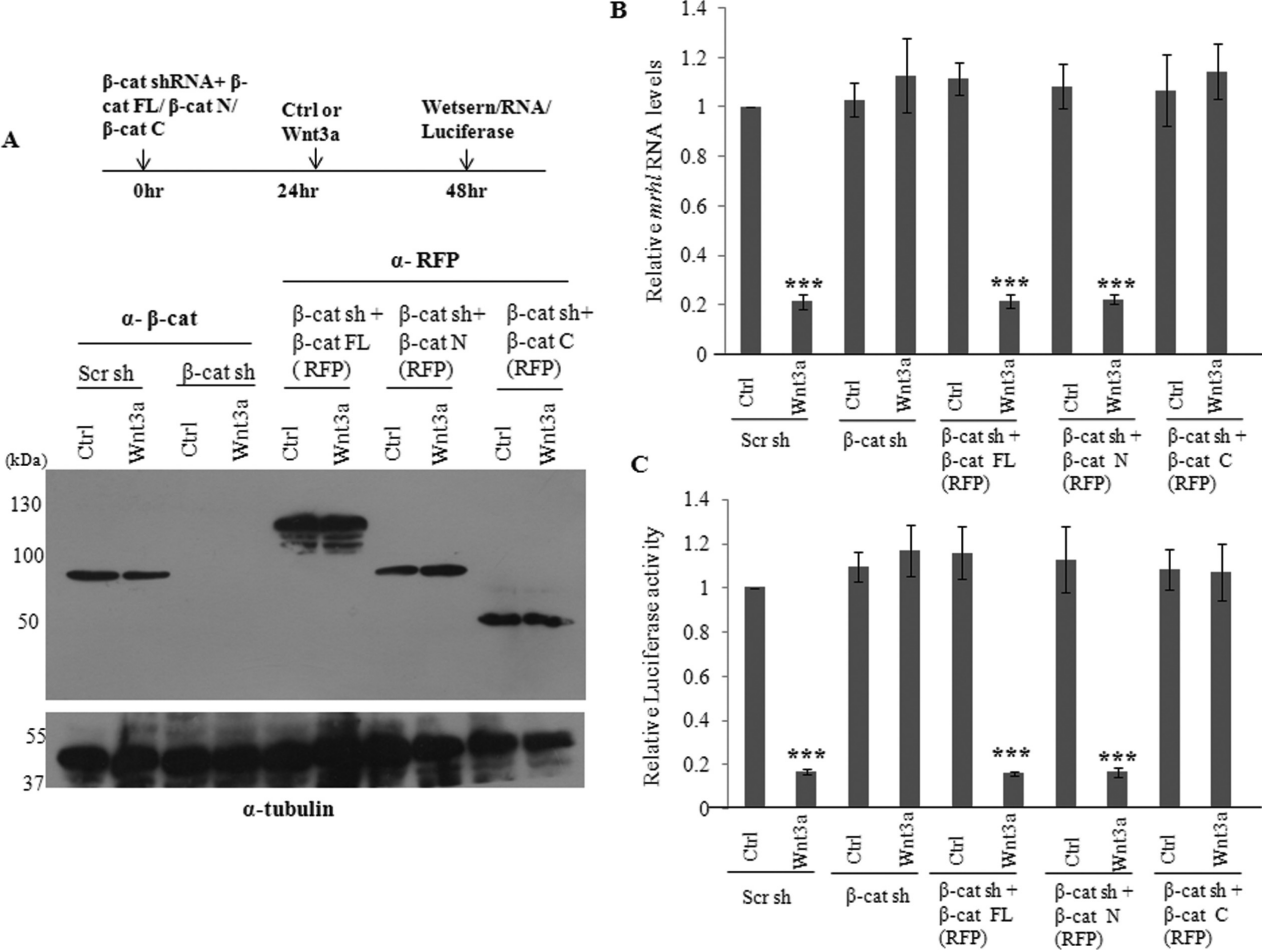
β-Catenin transactivation domain at the N-terminus is required for down regulation of *mrhl* RNA. (**A**) Western blot to analyze the reduced levels of β-catenin after silencing using shRNA targeting the 3′ UTR and the increased levels after over expression of RFP tagged full-length β-catenin, N-terminal transactivation domain of β-catenin and C-terminal transactivation domain. (**B** and **C**) Expression analysis of *mrhl* RNA (B) and Luciferase assay using Wild Type *mrhl* RNA promoter construct (C) upon treatment with shRNAs targeting the UTR region of β-catenin mRNA as well as after over expression of RFP tagged full length β-catenin, N-terminal transactivation domain of β-catenin and C-terminal transactivation domain. Data in (B) and (C) are plotted as mean ± SD, *n* = 4. ***P ≤ 0.0005 (*t*-test).

We also validated the necessity of β-catenin and TCF4 for the down regulation of mrhl RNA gene expression by using shRNA approach. There was a drastic reduction in the protein levels of β-catenin and TCF4 after transfection of the respective shRNA constructs in control medium as well as Wnt3a CM (Figure 3B). As seen in Figure 3C, cells treated with scrambled shRNA followed by Wnt3a CM treatment showed down regulation of *mrhl* RNA expression, but cells treated with either β-catenin or TCF4 shRNA followed by Wnt3a CM treatment failed to down regulate *mrhl* RNA expression (expression comparable to control medium treated cells). Luciferase reporter assay with the *mrhl* RNA promoter construct also showed significantly reduced reporter activity upon Wnt3a CM treatment while there was no reduction in the Luciferase activity upon treatment with β-catenin or TCF4 shRNA (Supplementary Figure S2A or S2A). In order to confirm the specificity of shRNA mediated knockdown experiments, we carried out the rescue of β-catenin and TCF4 expression. For this purpose we used shRNAs targeting the 3′ UTR of β-catenin and TCF4 mRNAs and carried out over expression of β-catenin and TCF4 protein using plasmid constructs containing the cDNA of β-catenin and TCF4. As seen in Figure 3D, there was a drastic down regulation of β-catenin and TCF4 protein levels on use of 3′ UTR targeting shRNAs as well as substantial over expression of β-catenin or TCF4 proteins on use of the over expression plasmid constructs. As observed previously, the expression of *mrhl* RNA was not down regulated (in control medium as well as Wnt3a CM) upon β-catenin or TCF4 shRNA treatment (Figure 3E). However, the rescue of the expression of β-catenin or TCF4 resulted in the down regulation of *mrhl* RNA expression in Wnt3a CM treated Gc1-Spg cells (Figure 3E). Similar result was obtained in the Luciferase reporter assay carried out after the rescue of β-catenin or TCF4 expression (Supplementary Figure S2B or S2B). We also carried out cycloheximide treatment of Gc1-Spg cells (in control medium as well as Wnt3a CM) to block the translation of β-catenin and TCF4 and then examine the expression levels of mrhl RNA. The protein levels of β-catenin and TCF4 were substantially reduced after 24 h and 48 h of cycloheximide treatment (Supplementary Figure S2C or S2C). *Mrhl* RNA levels were significantly down regulated upon 48 h of cycloheximide treatment only in Wnt3a CM treated Gc1-Spg cells (Supplementary Figure S2D or S2D). Further we examined whether the N-terminal or the C-terminal domain of β-catenin is required for the down regulation of *mrhl* RNA. We used RFP tagged constructs of full length β-catenin (β-cat FL-RFP), N terminal β-catenin (β-cat N-RFP, 1–576 amino acids), C-terminal β-catenin (β-cat C-RFP, 577–780 amino acids). Upon knock down of β-catenin using the 3′ UTR targeting shRNA, we over expressed β-cat FL-RFP, β-cat N-RFP and β-cat C-RFP (Figure 4A). Over expression of β-cat FL-RFP and β-cat N-RFP upon knock down of β-catenin brought about the down regulation of *mrhl* RNA expression (Figure 4B) and reduction in the Luciferase reporter activity (Figure 4C). This suggests that the N- terminal domain of β-catenin is required for the down regulation of *mrhl* RNA expression. Altogether these results conclusively demonstrate the requirement of β-catenin and TCF4 for the down regulation of *mrhl* RNA expression upon Wnt signaling activation and hence *mrhl* RNA gene can be identified as one of the Wnt signaling targets in mouse spermatogonial cells.

### Identification of Ctbp1 as the β-catenin associated co-repressor essential for the down regulation of *mrhl* RNA upon Wnt signaling activation

**Figure 5.**
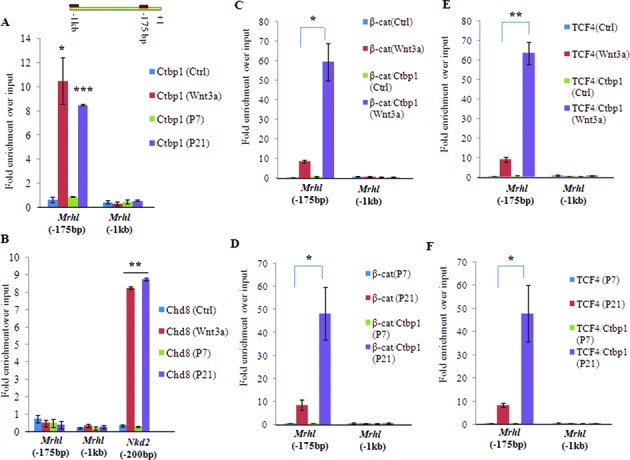
Ctbp1, but not Chd8 binds the *mrhl* RNA promoter at the TCF4 binding site [*Mrhl* (−175 bp)]. (**A** and **B**) Ctbp1 (A) and Chd8 (B) ChIP in Gc1-Spg cells (Ctrl, Wnt3a CM treated) and mouse testis (P7, P21) with Nkd2 as positive control for Chd8 occupancy. Ctbp1 but not Chd8 occupies *Mrhl*(−175bp) only upon Wnt signaling activation. (**C–F)** Sequential ChIP analysis showing the co occupancy of β-catenin and Ctbp1 (C, D) on *Mrhl*(-175bp) and co-occupancy of TCF4 and Ctbp1(E, F) on *Mrhl*(−175 bp) upon Wnt signaling activation. Data are plotted as mean ± SD, *n* = 4. ***P ≤ 0.0005, **P ≤ 0.005, *P ≤ 0.05 (*t*-test).

In addition to the recruitment of β-catenin at the TCF4 binding site within the promoters of Wnt target genes, the molecular mechanism envisages the recruitment of either co-activators or co-repressors at these sites (27). Recent studies have shown the role of two proteins namely Ctbp1 and Chd8 as the β-catenin associated co-repressors (30–33). We employed the candidate approach and examined the role of these two proteins as probable co-repressors of β-catenin/TCF4 mediated down regulation of *mrhl* RNA expression. We confirmed the expression of these two co-repressors in the P7 and P21 mice testes (Supplementary Figure S3 or S3). Towards this objective, we sought to examine the co-occupancy of Ctbp1 and β-catenin/TCF4 as well as Chd8 and β-catenin/TCF4 on the *mrhl* RNA promoter by ChIP and sequential ChIP (ChIP-Re ChIP) experiments. We observed that Ctbp1 occupied the *mrhl* RNA promoter element at *Mrhl*(−175 bp) (Figure 5A) while Chd8 did not (Figure 5B). To determine the co-occupancy of Ctbp1 and β-catenin/TCF4 at *Mrhl* (−175 bp) we carried out sequential ChIP experiments. It is now very well established that if two proteins co-occupy at a particular genomic site, in a quantitative context the fold enrichment (over input DNA) of the sequential ChIP is equal to the product of the fold enrichments of the individual ChIP (34). Our sequential ChIP experiments showed the co-occupancy of Ctbp1 and β-catenin at *Mrhl(−175 bp)* (Figure 5C and D) as well as Ctbp1 and TCF4 at *Mrhl*(−175 bp) (Figure 5E and F). This co-occupancy of Ctbp1, β-catenin and TCF4 at *Mrhl*(−175 bp) was seen only under the conditioned Wnt signaling activation (Wnt3a CM treated Gc1-Spg cells) and the P21 testicular chromatin.

**Figure 6.**
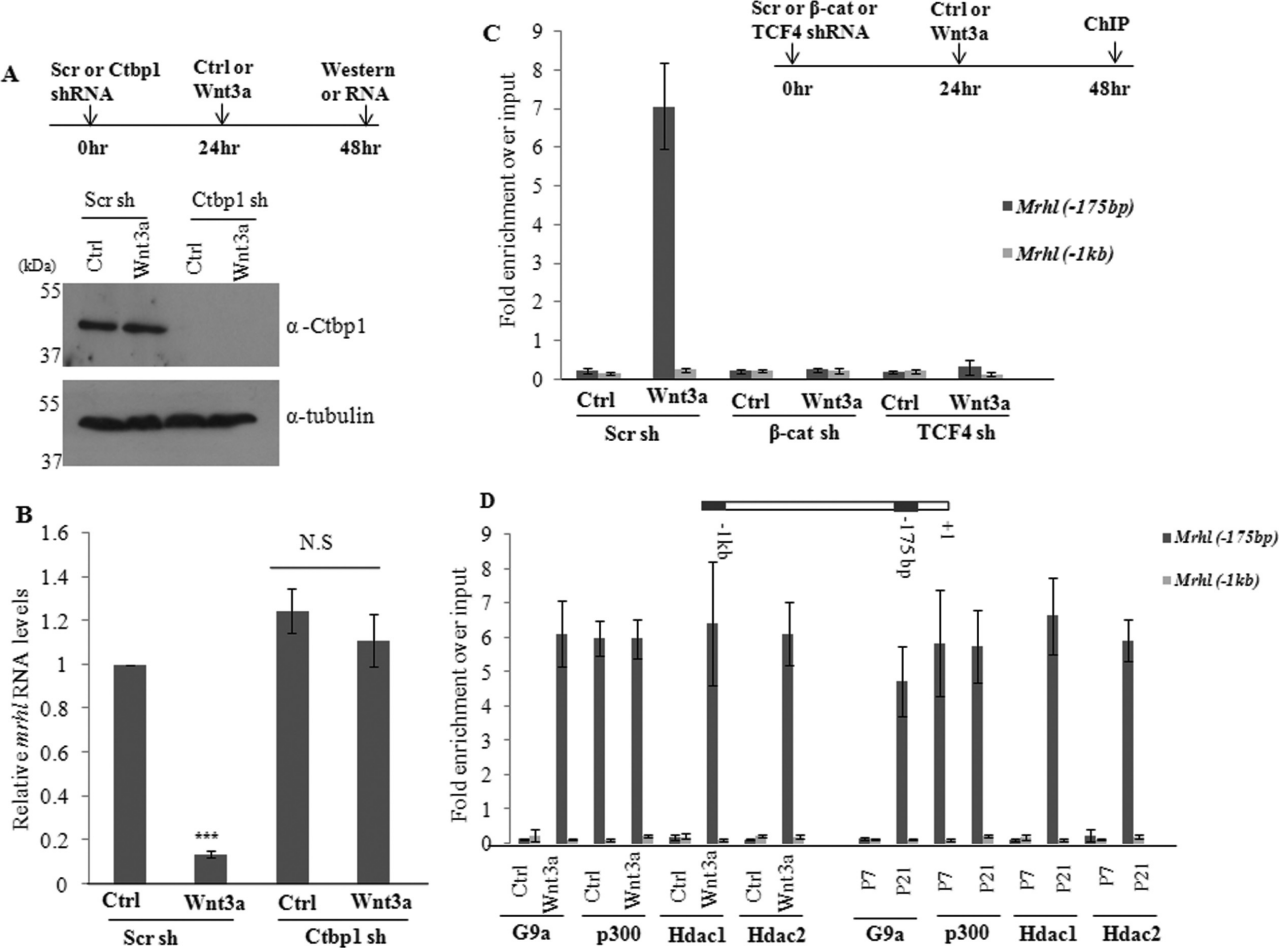
Ctbp1 functions as the co-repressor for *mrhl* RNA down regulation upon Wnt signaling activation. (**A**) Western blot showing significant down regulation of Ctbp1 upon shRNA treatment. (**B**) *Mrhl* RNA expression upon Ctbp1 down regulation. (**C**) Occupancy of Ctbp1 on *mrhl* RNA promoter upon down regulation of β-catenin or TCF4. (**D**) Occupancy of few of the known Ctbp1 associated proteins (G9a, p300, Hdac1 and Hdac2) on *mrhl* RNA promoter. Data are plotted as mean ± SD, *n* = 4. ***P ≤ 0.0005 (*t*-test). N.S.: not significant.

**Figure 7.**
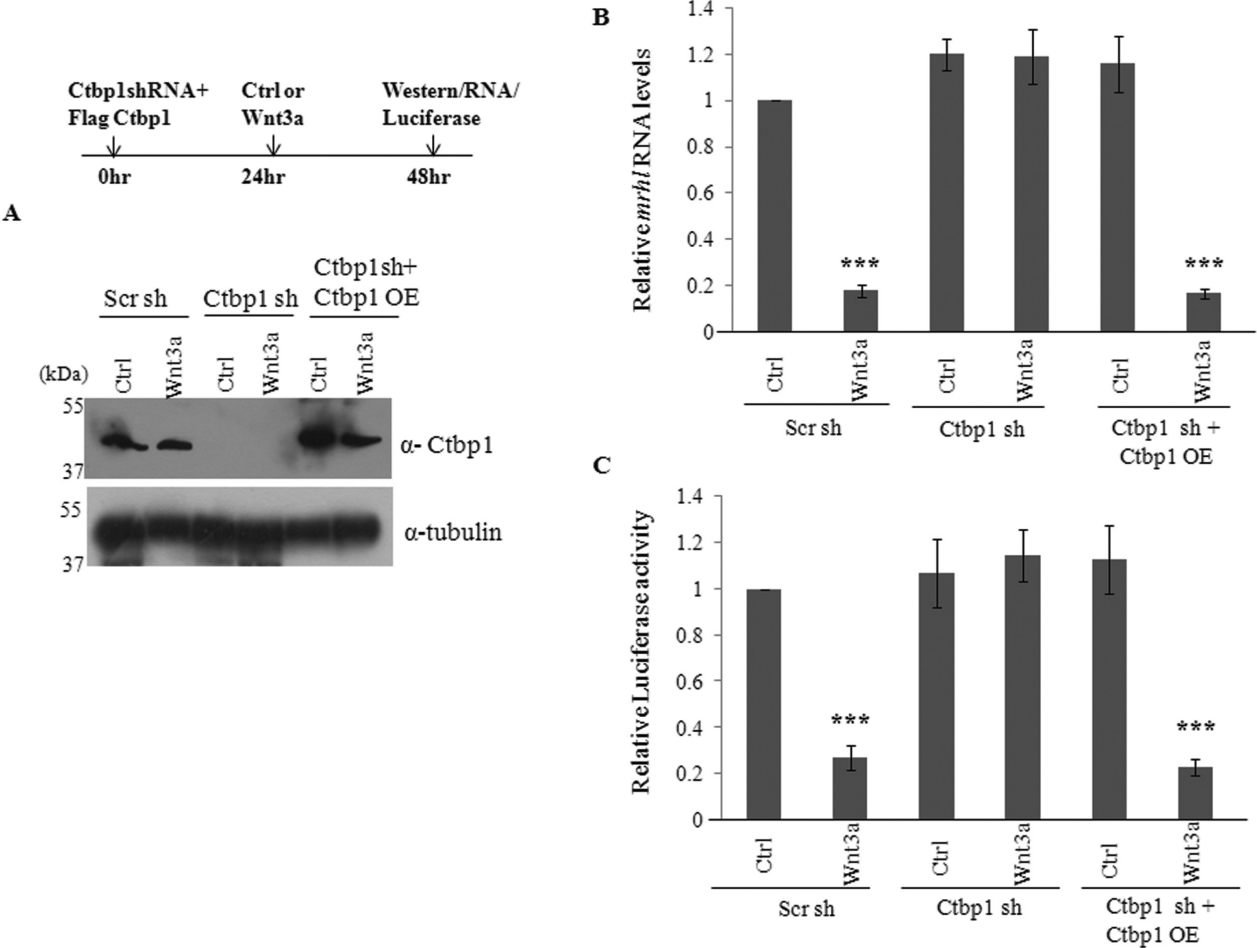
Exogenous expression of Ctbp1 suffices for repression of *mrhl* RNA. (**A**) Western blot to analyze the reduced levels of Ctbp1 after silencing using shRNA targeting the 3′ UTR and the increased levels of Ctbp1 after over expression of Ctbp1 protein. (**B** and **C**) Expression analysis of *mrhl* RNA (B) and Luciferase assay using wild-type *Mrhl* RNA promoter construct (C) upon treatment with shRNAs targeting the 3′ UTR region of Ctbp1 mRNA as well as after over expression of Ctbp1 protein. Data are plotted as mean ± SD, *n* = 4. ***P ≤ 0.0005 (*t*-test).

Since only Ctbp1 but not Chd8 occupied the promoter of *mrhl* RNA, we went ahead with silencing the expression of Ctbp1 in Gc1-Spg cells using the shRNA approach. Ctbp1 protein levels were drastically reduced upon Ctbp1 shRNA treatment in Gc1-Spg cells treated with control medium as well as Wnt3a CM (Figure 6A). Analysis of *mrhl* RNA expression revealed similar results to that of β-catenin and TCF4 silencing. *Mrhl* RNA was down regulated on Wnt3a CM treatment while the cells treated with Ctbp1 shRNA followed by Wnt3a CM treatment failed to down regulate the expression of *mrhl* RNA (expression level was the same as that in control cells) (Figure 6B). The occupancy of Ctbp1 on *Mrhl*(−175 bp) was regulated by β-catenin and TCF4 since the silencing of β-catenin and TCF4 led to loss of Ctbp1 occupancy at *Mrhl*(−175 bp) in Wnt3a CM treated Gc1-Spg cells (Figure 6C). It is known that Ctbp1 is part of a complex which contains many other proteins (35,36). Few of them include a histone methyl transferase G9a, histone acetyl transferase p300 and histone deacetylases Hdac1 and Hdac2. With a view to examine whether similar Ctbp1 complex may be present at the *mrhl* RNA proximal promoter region, we next checked for the occupancy of G9a, p300, Hdac1 and Hdac2 on the *mrhl* RNA gene promoter. G9a, Hdac1 and Hdac2 occupied *Mrhl*(−175 bp) proximal promoter region only upon Wnt signaling activation while p300 occupied *Mrhl*(−175 bp) even in the absence of Wnt signaling activation (Figure 6D). In a way similar to β-catenin and TCF4, we also used shRNA targeting the 3′ UTR of Ctbp1 mRNA followed by over expression of Ctbp1 protein. The efficiency of Ctbp1 knockdown as well as over expression are represented in Figure 7A. The expression of *mrhl* RNA was not down regulated (in control medium as well as Wnt3a CM) upon Ctbp1 shRNA treatment (Figure 7B). However, upon over expression of Ctbp1 in trans, *mrhl* RNA expression was down regulated in Wnt3a CM treated Gc1-Spg cells (Figure 7B). Similar results were obtained in the Luciferase reporter assay carried out after the over expression of Ctbp1 (Figure 7C). Therefore we conclude that Ctbp1 is the co-repressor for the down regulation of *mrhl* RNA upon Wnt signaling activation. We were also curious to see whether Ctbp1 interacts with β-catenin. Our one to one *in vitro* pull down experiments showed that they do not interact physically with each other (data not shown).

### Repressive histone modifications at the *mrhl* RNA promoter locus upon Wnt signaling activation

**Figure 8.**
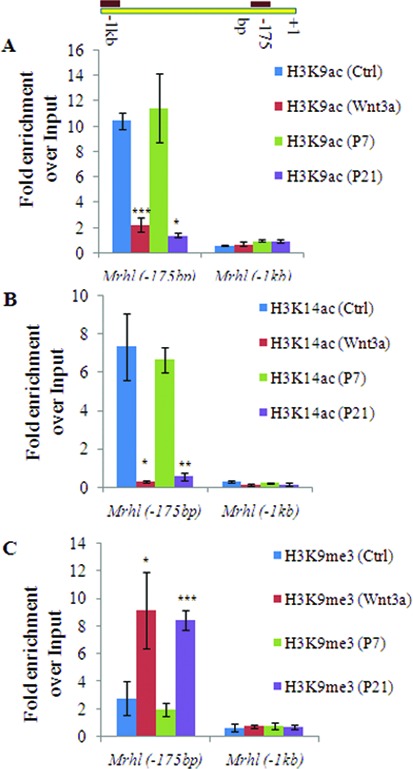
Changes in histone modification marks on the *mrhl* RNA promoter upon Wnt signaling activation. (**A** and **B**) Levels of H3K9ac (A) and H3K14ac (B) activation marks at *Mrhl*(−175 bp) in Gc1-Spg (Ctrl, Wnt3a) and mouse testis (P7, P21). Decrease in levels of H3K9ac and H3K14ac upon Wnt activation. (**C**) Analysis of H3K9me3 repression mark at *Mrhl*(−175 bp) in Gc1-Spg (Ctrl, Wnt3a) and mouse testis (P7, P21). Increase in level of H3K9me3 upon wnt activation. Data are plotted as mean ± SD, *n* = 4. ***P ≤ 0.0005, *P ≤ 0.05 (*t*-test).

Previous studies have uncovered the mechanism of Ctbp1 mediated transcriptional repression (35,36). As mentioned above, biochemical purification strategies have identified Ctbp1 as a part of a multimeric complex which contains many proteins that possess multiple histone modifying activities (34). Ctbp1 is majorly associated with three histone modifications namely deacetylation of H3K9, tri-methylation of H3K9 and deacetylation of H3K14 (35,37). We checked for these histone modification marks on the *mrhl* RNA promoter and also correlated them with the occupancy of Ctbp1 on the *mrhl* RNA promoter. We found that H3K9 acetylation (Figure 8A) and H3K14 acetylation (Figure 8B) showed a significant reduction while H3K9 tri methylation (Figure 8C) showed a marked increase at *Mrhl*(−175 bp) upon Wnt signaling activation (Wnt3a CM treated Gc1-Spg and P21 testis). In a control set of experiments, no such significant changes were observed at the *Mrhl* (−1 kb) promoter region for any of these histone modification marks. These changes in the histone modifications at *Mrhl*(−175 bp) correlated very well with the observation that Ctbp1 occupied *Mrhl*(−175 bp) only upon Wnt signaling activation.

### Wnt signaling induced *mrhl* RNA down regulation is required for up regulation of spermatogonial differentiation markers

**Figure 9.**
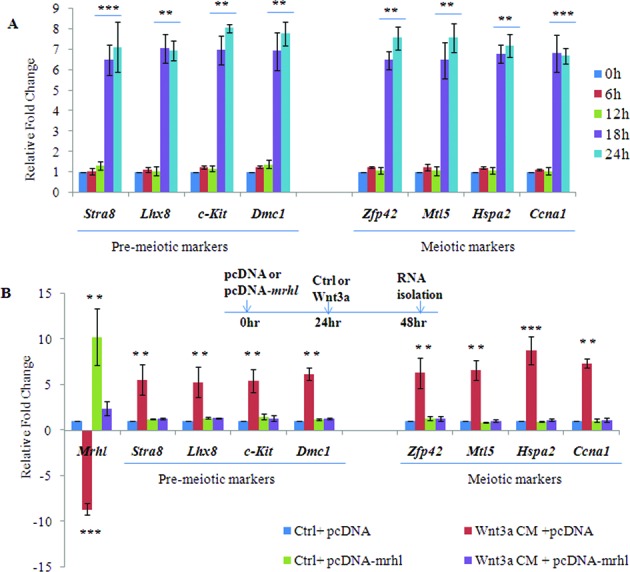
Requirement of Wnt signaling induced *mrhl* RNA down regulation for up regulation of differentiation markers. (**A**) Expression analysis of pre-meiotic markers (*Stra8, Lhx8, c-Kit* and *Dmc1*) as well as meiotic markers (*Zfp42, Mtl5, Hspa2* and *Ccna1*) in Gc1-Spg cells upon Wnt3a CM treatment for different time durations (0, 6, 12, 18 and 24 h). (**B** ) Expression analysis of pre-meiotic markers (*Stra8, Lhx8, c-Kit* and *Dmc1*) as well as meiotic markers (*Zfp42, Mtl5, Hspa2* and *Ccna1*) upon Wnt signaling activation as well as during over expression of *mrhl* RNA in trans (pcDNA-*mrhl*). Data are plotted as mean ± SD, *n* = 4. ***P ≤ 0.0005, **P ≤0.005 (*t*-test).

**Figure 10.**
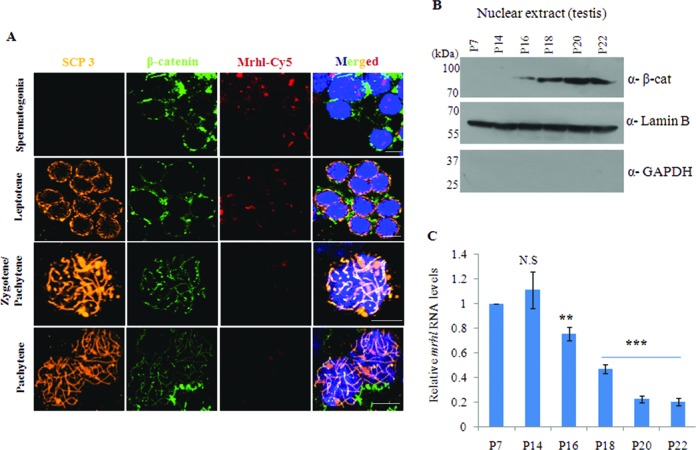
Down regulation of *mrhl* RNA coincides with nuclear localization of β-catenin during meiotic stages of mouse spermatogenesis *in vivo*. (**A**) Localization of β-catenin and *mrhl* RNA probed by immunofluorescence and RNA-FISH respectively during pre-meiotic (spermatogonia) and meiotic stages (Leptotene, Zygotene/Pachytene and Pachytene) of mouse spermatogenesis. Scp3 staining was used for staging the different meiotic prophase stages. (**B**) Western blot using nuclear lysates of mouse testis at different age (P7, P14, P16, P18, P20 and P22) in order to determine the timing of nuclear localization of β-catenin. Presence of Lamin B and absence of GAPDH shows the purity of nuclear lysates. (**C**) *Mrhl* RNA expression in mouse testis at different age (P7, P14, P16, P18, P20 and P22). Data plotted as mean ± SD, *n* = 4. ***P ≤ 0.0005, **P ≤ 0.005 (*t*-test). N.S.: not significant.

Gc1-Spg cells are derived from B-type spermatogonia which *in vivo* differentiate into meiotic spermatocytes in the mammalian testis. It is not clear at present which factors govern this commitment to differentiation *in vivo*. Based on the fact that Wnt signaling is activated in meiotic spermatocytes and our observation that *mrhl* RNA is down regulated in spermatocytes, we were curious to examine the Wnt3a ligand treated Gc1-Spg cells particularly with respect to the expression of differentiation markers. For this purpose, we assessed the transcript levels of few premeiotic markers (*c-Kit*, *Dmc1, Stra8* and *Lhx8*) and meiotic markers (*Zfp42, Hspa2, Mtl5* and *Ccna1*). Expression of c-Kit is increased in spermatogonia committed to meiotic prophase (38,39), Dmc1 is a meiosis specific recombinase expressed during first meiotic prophase (40,41), Stra8 is required for the early meiotic progression (42), while *Lhx8* expression has been shown to be increased in spermatogonia differentiating upon treatment with Kit ligand (38). Expression of Zfp42 and Mtl5 has been shown to be limited to spermatocytes (43,44), Ccna1 is known to be expressed in spermatocytes and essential for spermatocytes to enter in the first meiotic division (45,46) and Hspa2 is known to be required for completion of male germ cell meiosis (47). Gc1-Spg cells treated with Wnt3a CM showed a significant up regulation of all these 8 markers (Figure 9A). In order to determine whether the down regulation of *mrhl* RNA is a necessary event for the up regulation of these differentiation markers, we restored the levels of *mrhl* RNA by plasmid based over expression, in trans, and then scored for the expression of differentiation markers. When *mrhl* RNA was over expressed in Wnt3a CM treated cells, the expression of the differentiation markers was not up regulated (Figure 9B) (no significant change in the expression of the differentiation markers as compared to control treated cells). Over expression of *mrhl* RNA in control medium treated cells also did not lead to significant change in expression of differentiation markers. Interestingly we find from our previously reported microarray data of *mrhl* RNA down regulation in Gc1-Spg cells (GSE 19355) that there is no significant perturbation of expression of the differentiation markers under those conditions (Supplementary Figure S4 or S4). Therefore from this experiment we infer that the down regulation of *mrhl* RNA in the context of Wnt signaling activation is essential for regulating the expression of meiotic differentiation markers. At this juncture, we were curious to examine whether such an inverse relationship between the down regulation of *mrhl* RNA expression and β-catenin appearance in the nucleus (an indicator of wnt signaling activation) does reflect the *in vivo* situation. For this purpose, we performed RNA-FISH (RNA-fluorescent *in situ* hybridization) using LNA (locked nucleic acid) probes on the meiotic spreads prepared from mice testes to examine the expression and localisation of mrhl RNA at different meiotic prophase stages. SCP3 (synaptonemal complex protein 3) localization pattern along the chromosomes was used to identify cells of different stages of meiotic prophase namely leptotene, zygotene/pachytene and pachytene in the meiotic spreads while β-catenin localization was used to assess the Wnt signaling activation. As can be seen from Figure 10A, we could observe the nuclear localisation of β-catenin in the zygotene/pachytene and pachytene stage (as marked by SCP3 staining) which was also accompanied by significantly reduced levels of *mrhl* RNA. This observation agrees with the recent report of Kerr *et al*. demonstrating the nuclear localisation of β-catenin in pachytene spermatocytes (24). The correlation of *mrhl* RNA expression, β-catenin nuclear localization and meiotic progression was also substantiated by western Blot analysis using testicular nuclear lysates of mice of different ages (P7, P14, P16, P18, P20 and P22). Day 7 (P7) mouse testis contains spermatogonia as the predominant germ cells while at day 14 (P14) early spermatocytes are present (24). As seen in Figure 10B, β-catenin nuclear localization was seen from P16 to P22. Presence of Lamin B and absence of GAPDH confirmed the purity of the nuclear lysates. Also, the down regulation of *mrhl* RNA expression was seen from P16 to P22 (Figure 10C) thus strengthening our findings obtained using the Gc1-Spg cell line.

## DISCUSSION

Mammalian spermatogenesis is a unique process of cellular differentiation encompassing three different phases namely (i) mitotic phase, generating more of spermatogonial cells, (ii) meiotic phase, generating genetically diverse haploid spermatids and (iii) spermiogenesis, involving maturation of spermatids to sperms that includes nuclear reshaping and chromatin packaging (48,49). Wnt signaling is a highly conserved signaling pathway playing a significant role in development and disease. Various groups have studied the significance of Wnt signaling in postnatal testicular function and now we know that Wnt signaling is essential at multiple stages of spermatogenesis (24,50–53). Increasing number of reports show the involvement of lncRNAs as crucial players in the regulation of cellular differentiation, development and disease, few lncRNAs are shown to be regulated by Wnt signaling (54–56). In one of our earlier studies, we had reported that *mrhl* lncRNA negatively regulates Wnt signaling in mouse spermatogonial cells (21). In this report, we have demonstrated that *mrhl* RNA is responsive to Wnt signaling activation as *mrhl* RNA gets down regulated upon Wnt3a treatment in B-type spermatogonial (Gc1-Spg) cells. As per our knowledge, this is the first report of a lncRNA being regulated by Wnt signaling pathway. Although there are few reports of lncRNA as targets of Wnt signaling, the detailed molecular mechanism(s) involved in their regulation has not been studied. The present study describes the molecular events leading to down regulation of *mrhl* RNA upon Wnt signaling in mouse spermatogonial cells.

*Mrhl* RNA is an intronic lncRNA transcribed from the 15th intron of mouse *phkb* gene as an independent transcription unit and the proximal promoter region contains many putative transcription factor binding sites including that of transcription factor TCF4. The mechanism of *mrhl* RNA down regulation is executed mainly at this TCF4 binding site. The requirement of beta catenin along with TCF4 for the down regulation of *mrhl* RNA only upon activation of Wnt signaling further qualifies *mrhl* as a Wnt target gene. Further, the requirement of a co-repressor in form of Ctbp1 accompanied by a repressive histone modification signature (reduced H3K9ac and H3K14ac and increased H3K9me3) at the *mrhl* RNA proximal promoter fits in nicely with the very well documented modes of regulation of gene expression as a consequence of Wnt signaling activation. Ctbp1 has been shown by Shi *et al*. to be a part of a multimeric complex which consists of proteins with multiple histone modifying activities and propose a repressive mode of action for the Ctbp complex which is coordinated by the histone modifications catalyzed by Hdac1/2 and G9a (35). Ctbp1 was initially reported by Valenta *et al*. to function as a repressor of the Wnt target gene *Axin2* in HEK 293 cells by its interaction with TCF4 (30). But, in their subsequent study, they could not show any association or direct interaction between Ctbp1 and TCF4 (57). Similarly, Hamada and Bienz also could not detect any physical or functional interaction of Ctbp1 and TCF4 but clearly demonstrated that Ctbp can antagonize canonical Wnt signaling (58). The repression of *Axin2* by Ctbp1 was shown to be alleviated by Trichostatin A treatment indicating dependence on the activity of histone deacetylase. In another study, detailed mechanism of Ctbp1 mediated repression has been delineated by Stossi *et al*. in the context of repression of early estrogen-repressed genes by Estrogen Receptor Alpha (ERα) (37). In their study, they conclude that ERα uses Ctbp1 in transcriptional repression and p300 partners with Ctbp1 when gene repression is required. After recruitment of Ctbp1, the histone acetyl transferase activity of p300 is inhibited by Ctbp1, which leads to repressive histone modification marks (reduction of H3K14ac, H3K9ac and increase in H3K9me3) and ultimately gene repression. Overall our findings like association of Ctbp1 with β-catenin/TCF4, requirement of Ctbp1 for *mrhl* RNA down regulation and binding of known Ctbp1 associated proteins like G9a, Hdac1, Hdac2 and p300 on the *mrhl* RNA promoter along with increased repressive histone modification marks support the notion that Ctbp1 functions as a co-repressor for *mrhl* RNA down regulation in a much similar way as have been reported before. It is not surprising that the regulation of a lncRNA expression (here *mrhl* RNA) which is a RNA polymerase II transcribed gene employs similar mechanism for regulation as has been established for many of the other protein coding genes. It is pertinent to point out here that Wnt signaling although brings about down regulation of the *mrhl* gene; the transcription of *phkb* gene is not perturbed. It would be interesting to study in future how the transcription is differentially regulated at a parent gene and a gene encoded within an intron of the parent gene.

**Figure 11.**
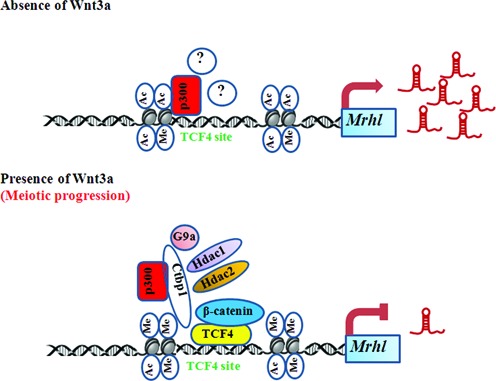
Model summarizing the findings of present study. The model depicts the changes occurring at the proximal promoter region of *mrhl* RNA at the TCF4 binding site upon Wnt signaling activation with respect to the binding of different proteins like β-catenin, TCF4, Ctbp1 and Ctbp1 associated proteins (p300, G9a, Hdac1 and Hdac2). p300 binds at the TCF4 binding site even in the absence of Wnt3a and other proteins (?) could be associated with p300 for regulation of *mrhl* RNA expression. The changes in histone modifications are also shown. In the presence of Wnt3a, *mrhl* RNA down regulation possibly leads to meiotic commitment and differentiation.

In most of our experiments, we have also used mouse testicular tissue chromatin as an *in vivo* correlate for our experiments performed in a cell culture system. In our recent report, where we have studied the role of *mrhl* RNA in the regulation of gene expression at the level of chromatin we showed a perfect correlation of cell culture system and mouse testicular tissue, proving the biological relevance of *mrhl* RNA mediated gene regulation (22). *Mrhl* RNA is also down regulated in P21 testis (predominantly spermatocytes, Wnt activated state) as compared to P7 testis (predominantly spermatogonia, Wnt repressed state). In this context, an inverse correlation between the expression status of *mrhl* RNA and Wnt signaling raises an important biological question as to whether Wnt signaling regulates the meiotic commitment and differentiation of B type spermatogonia into meiotic spermatocytes and what is the role of *mrhl* RNA if any in this differentiation process. In these experiments we also observed that the β-catenin staining of zygotene/pachytene spermatocytes (Figure 10A) was more along the entire axis of the chromosomes, the significance of which cannot be ascertained at present. In addition to these observations our preliminary experiments described in the present study further show that various meiotic differentiation markers are indeed up regulated in Gc1-Spg cells following Wnt3a ligand treatment. More importantly, we observed that over expression of *mrhl* RNA, in trans, abrogated the up regulation of these meiotic differentiation markers, showing the necessity of Wnt signaling induced *mrhl* RNA down regulation for the differentiation. A model summarizing our findings reported in the present study has been depicted in Figure 11. We would like to stress here that just the down regulation of *mrhl* RNA alone is not sufficient for up regulation of meiotic markers expression as revealed from our microarray data. Hence, we would like to believe that Wnt signaling triggers several cascading events in order to initiate meiotic commitment and in this process down regulation of *mrhl* RNA expression is a critical event. Thus a detailed analysis of signaling events leading to commitment of spermatogonia towards differentiation into meiotic prophase spermatocytes would throw much light in our understanding of this very important biological phenomenon and more particularly, the role of *mrhl* RNA in this process. It is also worth mentioning here that the role of *mrhl* RNA in meiotic commitment of spermatogonial cells may be only one of its several biological roles, since the down regulation of *mrhl* RNA alters several other genes not associated with Wnt signaling pathway and also occupies several chromatin loci that are not connected with Wnt signaling (21,22).

## Supplementary Material

SUPPLEMENTARY DATA
