# Supplementary material for: Mechanism of Wnt signaling induced down regulation of mrhl long non-coding RNA in mouse spermatogonial cells
Source: Nucleic Acids Res. 2015 Oct 7;44(1):387–401. doi: 10.1093/nar/gkv1023 (PMC4705645; doi:10.1093/nar/gkv1023)
Supplement: SUPPLEMENTARY DATA [file supp_44_1_387__index.html]

Mechanism of Wnt signaling induced down regulation of mrhl long non-coding RNA in mouse spermatogonial cells — Mechanism of Wnt signaling induced down regulation of mrhl long non-coding RNA in mouse spermatogonial cells — SUPPLEMENTARY DATA 

# Mechanism of Wnt signaling induced down regulation of *mrhl* long non-coding RNA in mouse spermatogonial cells

## SUPPLEMENTARY DATA

- SUPPLEMENTARY DATA
- SUPPLEMENTARY DATA
- SUPPLEMENTARY DATA
- SUPPLEMENTARY DATA
- SUPPLEMENTARY DATA
- SUPPLEMENTARY DATA
