# Supplementary material for: Mechanism of Wnt signaling induced down regulation of mrhl long non-coding RNA in mouse spermatogonial cells
Source: Nucleic Acids Res. 2015 Oct 7;44(1):387–401. doi: 10.1093/nar/gkv1023 (PMC4705645; doi:10.1093/nar/gkv1023)
Supplement: SUPPLEMENTARY DATA [file supp_gkv1023_nar-02298-v-2015-File013.docx]

**Supplementary Figure Legends**

**Supplementary Figure 1 (S1). (A)** Localization of β-catenin using immunofluorescence in Gc1-Spg cells treated with Wnt3a CM for different time durations (0, 6, 12, 18 and 24hr). **(B)** Western blot analysis (using total cell lysate) for levels of Phospho (S9) Gsk-3β and Phospho (S33/S37/T41) β-catenin upon Wnt3a CM treatment in Gc1-Spg cells. α- tubulin is used as a loading control. **(C)** Localization of β-catenin using immunofluorescence in HEK293 cells treated with Wnt3a CM for different time durations (0, 3, 6, 9 and 12hr).

**Supplementary Figure 2 (S2). (A)** Luciferase assay in Gc1-Spg cells upon silencing of β-catenin or TCF4 and treated with control medium or Wnt3a CM using plasmid constructs containing 1kb upstream promoter region of *mrhl* RNA gene with wild type TCF4 binding site. **(B)** Luciferase assay in Gc1-Spg cells upon treatment with shRNAs targeting the 3’UTR region of β-catenin or TCF4 mRNA as well as after over expression/rescue of β-catenin or TCF4 protein. **(C)** Treatment of Gc1-Spg cells with Cycloheximide (100µg/ml) for different time durations (0, 6, 12, 24 and 48 hr) and analysis of protein levels of β-catenin, TCF4 and α- tubulin. **(D)** Expression analysis of *mrhl* RNA after treatment of Gc1-Spg cells with Cycloheximide (100µg/ml) for 48 hr and control medium or Wnt3a CM for 24 hr. Data in A),B) and D) are plotted as Mean ± SD, n=4. *** P≤ 0.0005 (*t* test).

**Supplementary Figure 3 (S3).** Western Blot showing the expression of the co repressors Chd8 and Ctbp1 in P7 and P21 mice testis.

**Supplementary Figure 4 (S4).** Expression analysis of the pre-meiotic markers (*Stra8, Lhx8, c-Kit* and *Dmc1*) as well as meiotic markers (*Zfp42, Mtl5, Hspa2* and *Ccna1*) in Gc1-Spg cells upon down regulation of *mrhl* RNA (data extracted from GSE 19355).
