# Supplementary figures and images for: Mechanism of Wnt signaling induced down regulation of mrhl long non-coding RNA in mouse spermatogonial cells
Source: Nucleic Acids Res. 2015 Oct 7;44(1):387–401. doi: 10.1093/nar/gkv1023 (PMC4705645; doi:10.1093/nar/gkv1023)

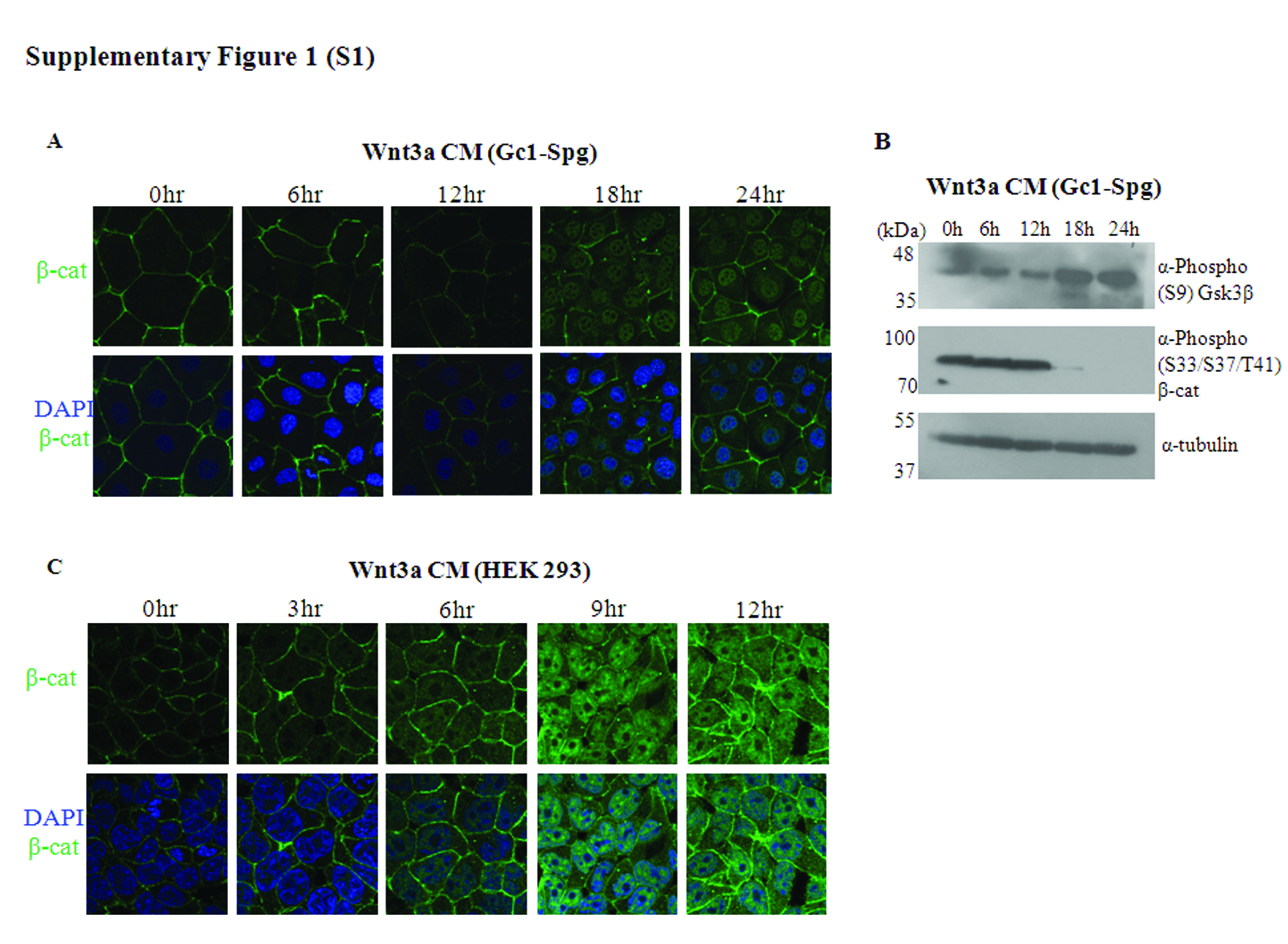

Supplement: SUPPLEMENTARY DATA [file supp_gkv1023_nar-02298-v-2015-File014.tif]

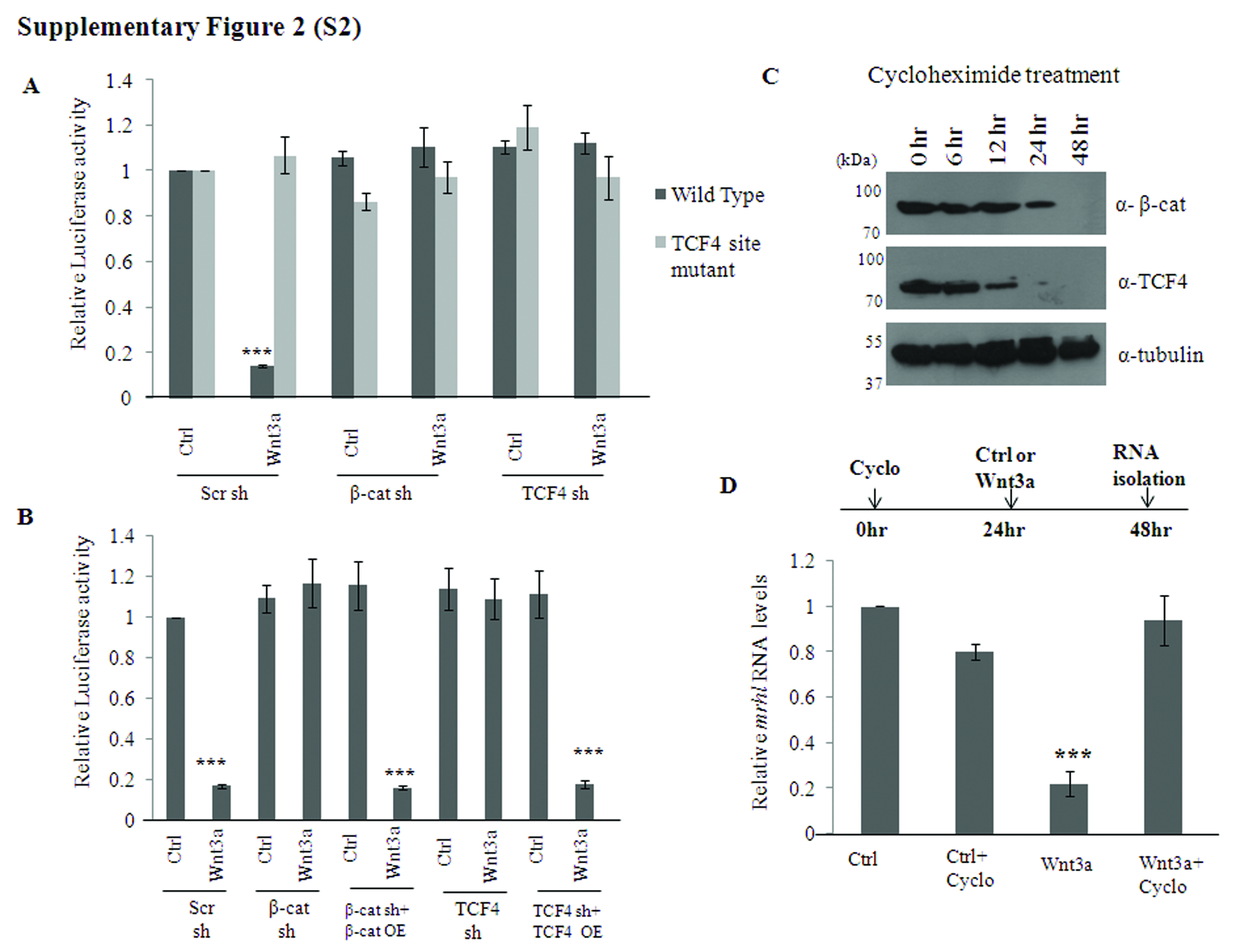

Supplement: SUPPLEMENTARY DATA [file supp_gkv1023_nar-02298-v-2015-File015.tif]

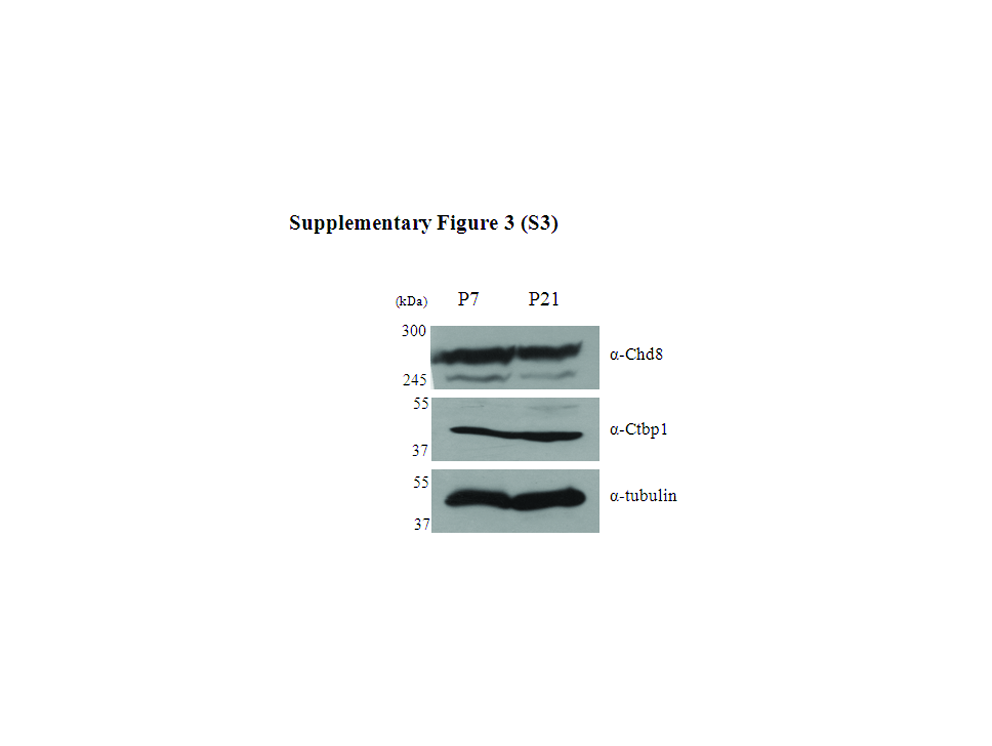

Supplement: SUPPLEMENTARY DATA [file supp_gkv1023_nar-02298-v-2015-File016.tif]

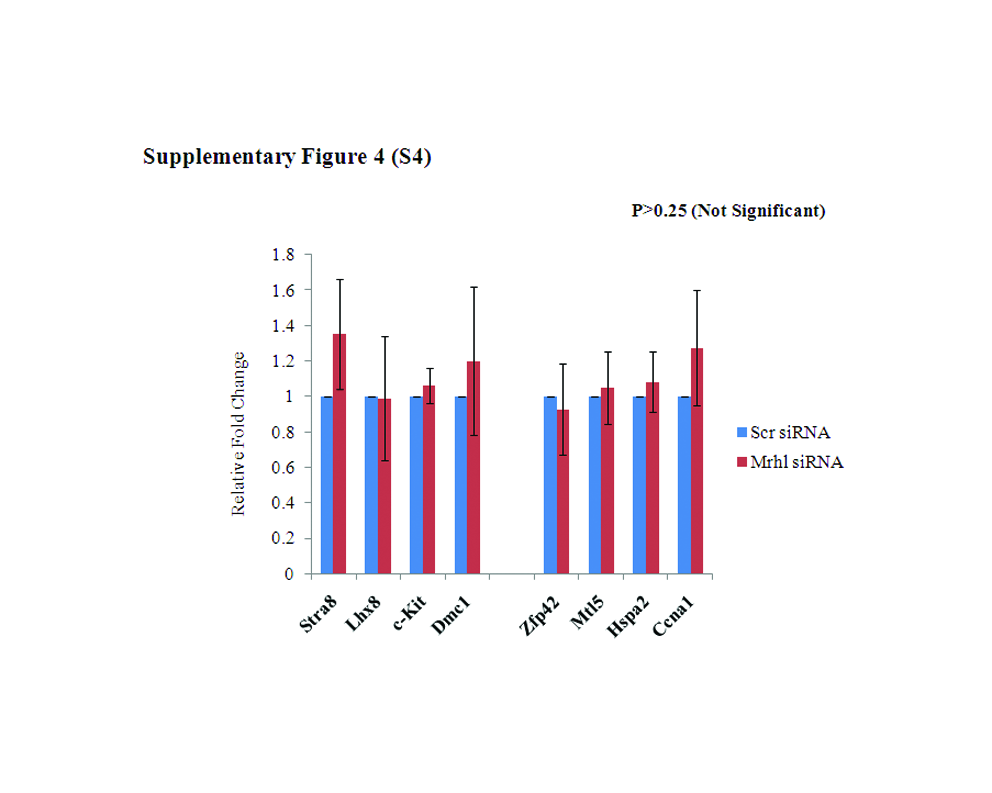

Supplement: SUPPLEMENTARY DATA [file supp_gkv1023_nar-02298-v-2015-File017.tif]
